# Conceptual Framework for Community-Based Prevention of Brown Dog Tick–Associated Rocky Mountain Spotted Fever

**DOI:** 10.3201/eid3011.240293

**Published:** 2024-11

**Authors:** Maureen K. Brophy, Erica Weis, Naomi A. Drexler, Christopher D. Paddock, William L. Nicholson, Gilbert J. Kersh, Johanna S. Salzer

**Affiliations:** Centers for Disease Prevention and Control, Atlanta, Georgia, USA (M.K. Brophy, N.A. Drexler, C.D. Paddock, W.L. Nicholson, G.J. Kersh, J.S. Salzer); Kapili Services, Honolulu, Hawaii, USA (E. Weis).

**Keywords:** Rocky Mountain spotted fever, ticks, One Health, health equity, vector-borne disease, tick-borne disease, brown dog tick, bacteria, *Rickettsia rickettsii*, *Rhipicephalus sanguineus*, zoonoses, *Dermacentor* species, United States, Mexico

## Abstract

Rocky Mountain spotted fever (RMSF) is a severe tickborne disease that can reach epidemic proportions in communities with certain social and ecologic risk factors. In some areas, the case-fatality rate of brown dog tick-associated RMSF is up to 50%. Because of the spread of brown dog tick–associated RMSF in the southwestern United States and northern Mexico, the disease has the potential to emerge and become endemic in other communities that have large populations of free-roaming dogs, brown dog ticks, limited resources, and low provider awareness of the disease. By using a One Health approach, interdisciplinary teams can identify communities at risk and prevent severe or fatal RMSF in humans before cases occur. We have developed a conceptual framework for RMSF prevention to enable communities to identify their RMSF risk level and implement prevention and control strategies.

Rocky Mountain spotted fever (RMSF) is the deadliest tickborne disease in the Western Hemisphere. RMSF is caused by the bacterium *Rickettsia rickettsii*, which is primarily transmitted to humans by *Dermacentor* spp. ticks in the United States. RMSF exposures associated with the brown dog tick (*Rhipicephalus sanguineus* sensu lato) are different from those associated with *Dermacentor* spp. ticks. For brown dog tick–associated RMSF, the primary site of exposure is in the peridomestic environment, which is in and around homes. Circulation of the bacteria in the peridomestic environment may go unnoticed until there is a severe case or death. During July–November 2023, there were 5 confirmed cases of RMSF and 3 deaths in southern California, USA, all in people who had traveled to or resided in Tecate, Baja California, Mexico, where there is high incidence of RMSF associated with brown dog ticks (*1*,*2*). Those cases suggest the introduction of the pathogen to new locations is not only possible but likely. The travel-associated cases in California, along with recent emergence in Arizona, USA, and reemergence in Mexico, suggests that RMSF might occur in other global areas with similar community risk factors.

## Brown Dog Tick–Associated RMSF in Mexico

Whereas cases of RMSF associated with brown dog tick transmission were not conclusively identified in the United States until 2005 (*3*), medical reports of a lethal illness described as a petechial rash and malignant scarlet fever in Mexico date back to 1903 (*4*). In 1943, epidemiologists identified this illness as the same RMSF that had been discovered in the United States around the turn of the 20th Century (*5*,*6*). They described the clinical manifestations and epidemiology of the disease and experimentally confirmed the association with brown dog ticks. Experimental studies in previous publications showed brown dog ticks were an efficient vector of *R. rickettsii* through 2 generations before this epidemiologic linkage (*7*). During 1918–1943, medical records indicate that >200 cases of RMSF occurred throughout Mexico, often clustered in neighborhoods or within households (*4*). The case clustering is a frequently observed characteristic of brown dog tick–associated RMSF transmission because the tick lives in and around human dwellings to be near its preferred host, domesticated dogs. A reduction in cases was reported after the 1940s, and whereas the reason for the decline is unknown, there is a possible connection with high use of DDT to control malaria in endemic regions throughout Mexico and the United States (*8*). However, RMSF has resurged in northern Mexico; 1,394 cases and 247 deaths were reported during 2003–2016, and the case-fatality rate was 18%, higher than previously seen (*4*,*9*). The resurgence of RMSF in Mexico has been particularly evident in the states of Sonora and Baja California but includes many border states in the northern part of the country, such as Chihuahua, Coahuila, and Nuevo Leon (*10*–*14*).

## Brown Dog Tick–Associated RMSF in Arizona

RMSF was historically rare in Arizona, consisting of only sporadic cases primarily associated with travel to endemic areas, until September 2003, when a fatal case occurred in an infant with no history of travel outside of their Indigenous Nation (*3*,*15*). During the investigation, 16 additional cases were identified in the original community and a neighboring Indigenous Nation; the earliest recognized case had occurred in 2002 (*3*). The typical vectors, *Dermacentor andersoni* or *D. variabilis* ticks, were absent, but many brown dog ticks on dogs secured on the property, free-roaming, and in the peridomestic environment were found. Of the brown dog ticks tested, 3% were positive for *R. rickettsii* (*3*). Implementation of intensive prevention measures reduced tick populations and temporarily halted cases. RMSF became endemic in the 2 communities, averaging 5–10 cases per year, with a case-fatality rate of ≈11% (*16*). During 2009–2011, cases of RMSF were confirmed on 4 other Indigenous Nations in Arizona. Those additional communities rapidly implemented prevention measures and have been able to reduce or eliminate additional human cases of RMSF to date.

Brown dog tick–associated RMSF emergence has historically been met with reactionary public health action, such as increased surveillance and intervention occurring after human cases are identified. However, evidence of *R. rickettsii* circulation in a zoonotic cycle before human cases were identified has been documented in countries including Brazil (*17*,*18*), Panama (*19*), and Costa Rica (*20*,*21*), suggesting early intervention could prevent the spread of RMSF to the human population. Within the United States, conditions for RMSF emergence are already present in some communities. A recent study found *R. rickettsii* in 1 of 10 adult and 1 of 20 larval brown dog ticks tested from Palm Beach, Florida, USA (*22*), where this tick species has not been reported to spread RMSF to date. In regions across the globe with similar suitable climates (i.e., 20°C–35°C and relative humidity 35%–95%) (*23*), if brown dog ticks carrying *R. rickettsii* were to infest free-roaming dogs or those with limited access to veterinary care, local circulation could follow and lead to outbreaks within the human population.

Local public health agencies should remain vigilant in monitoring for RMSF transmission, especially in regions with a suitable climate for brown dog ticks, large populations of free-roaming dogs, and limited access to medical and veterinary services. Identifying areas of high risk for brown dog tick–associated RMSF is necessary because mitigation efforts are expensive in terms of financial and human resource investment. Developing a conceptual framework for RMSF prevention can aid in identifying communities of high risk and implementing an early warning system that incorporates acarological surveillance. This early warning will increase preparedness and protect human lives while ensuring limited resources are appropriately allocated. This framework requires a One Health approach with expertise from medical, veterinary, and vector control professionals within each community (*9*,*24*,*25*).

## Theoretical Framework

**Figure 1:**
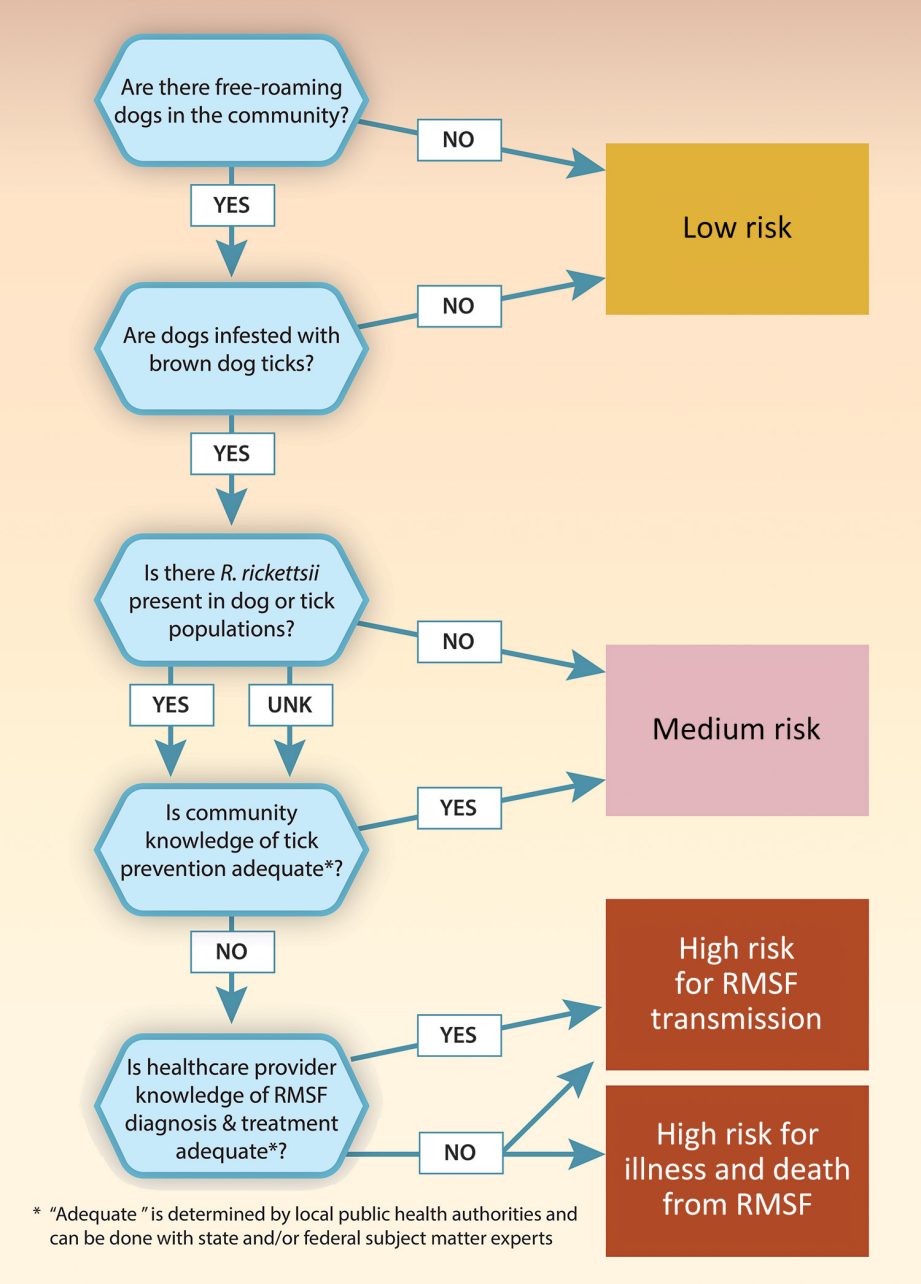
Community risk assessment for brown dog tick–associated RMSF. Communities with free-roaming dogs, high levels of brown dog ticks, and Rickettsia rickettsii in the dog or tick population are considered medium risk for RMSF transmission. Communities with those factors as well as inadequate community knowledge of tick prevention are considered high risk for RMSF transmission. If healthcare provider knowledge of RMSF diagnosis and treatment is also inadequate, the community is also considered high risk for severe illness or death from RMSF. RMSF, Rocky Mountain spotted fever.

Preventing RMSF from emerging in a community at risk is necessary because it is difficult to eliminate the disease once it becomes endemic. We have provided a basic framework of risk factors (Figure 1) and outlined the indicators and action items established by an Arizona state interdisciplinary coalition, which is currently in use by Arizona communities, to assess the risk for brown dog tick–associated RMSF (Table). We believe that if a community has free-roaming dogs, high levels of brown dog ticks, and inadequate medical and veterinary care, there is a medium risk for emergence of RMSF. If *R. rickettsii* is established in the tick or dog population and there are gaps in community understanding and application of tickborne disease prevention, gaps in healthcare worker knowledge of RMSF diagnosis and treatment, or a combination of any of those factors, there is a high risk for emergence. Communities without free-roaming dogs and without high levels of brown dog ticks are considered low risk.

**Table:** Conceptual framework for community-based prevention of RMSF listing proposed indicators and action items for communities at medium and high risk for endemic transmission*

Core function	Recommended risk level indicators	Action	Relative cost
Healthcare system coordination and public health reporting	Presence or absence of provider education around ticks and tickborne diseases, provider understanding of diagnosing and treating tickborne diseases, diagnostic testing capacity, distance to healthcare, availability of medical transport	Implement standardized RMSF patient treatment protocol in all affected areas to include follow up contact to ensure treatment continues if the patient leaves endemic area health facilities	$
		Use RMSF patient treatment algorithm in patients experiencing with fever or a history of contact with ticks	$$
		Disseminate education on RMSF for support staff and healthcare providers	$
		Require continuing medical education for healthcare providers, including MDs in primary care, emergency care, internal care, family practice, and pediatrics, physician assistants, and nursing staff providing care; consider embedding RMSF training course into the onboarding process for new hires	$$
		Establish a clinical task force to address areas of varying needs and priorities	$
Community education and outreach	Percent of population below local poverty level, educational attainment in community; presence or absence of health education around ticks and tickborne diseases; knowledge, attitudes, and behaviors about personal and home tick prevention; percent of population with internet access	Disseminate education on RMSF for support staff and community health workers	$$
		Consider embedding RMSF training course into the onboarding process for new hires in public interfacing agencies	$
		Create an RMSF communication plan so all communities get consistent messaging	$
Animal control and veterinary programs	Wellness: presence or absence of veterinary services, availability of effective ectoparasite treatments for dogs in community, number of spayed or neutered animals, cost of effective ectoparasite treatments for dogs in community; free-roaming population status: density of free-roaming dogs, presence or absence of ordinance forbidding free roaming dogs, fencing and tethering behaviors across community; access to resources: presence or absence of animal control department, presence or absence of animal shelter space, number of low- or no-cost spay and neuter clinics	Establish animal control programs	$$$
		Establish veterinary services	$$$
Environmental tick surveillance and control	Harborage: presence or absence of municipal and community solid waste removal, landfill cost and availability; pesticide use: presence or absence of vector control program, presence or absence of certified pesticide applicators; community knowledge, Attitudes, and Practices about tickborne diseases: presence or absence of community education around ticks and tickborne diseases	Develop programs to provide regular tick control services for each home in affected areas	$$$
		Implement environmental tick surveillance to provide measurement and direction for prevention efforts	$$$
Finance and budget	Presence or absence of dedicated annual jurisdictional funding to all partners for RMSF prevention, presence or absence of personnel capable of writing and managing grants	Engage leadership to advocate for sustainable funding for all RMSF prevention partners	$
		Train personnel across all RMSF prevention partnering agencies in grant writing and management	$$

*Relative cost scale: $, lowest cost activities; $$, median cost activities; $$$, highest cost activities. RMSF, Rocky Mountain spotted fever.

**Figure 2:**
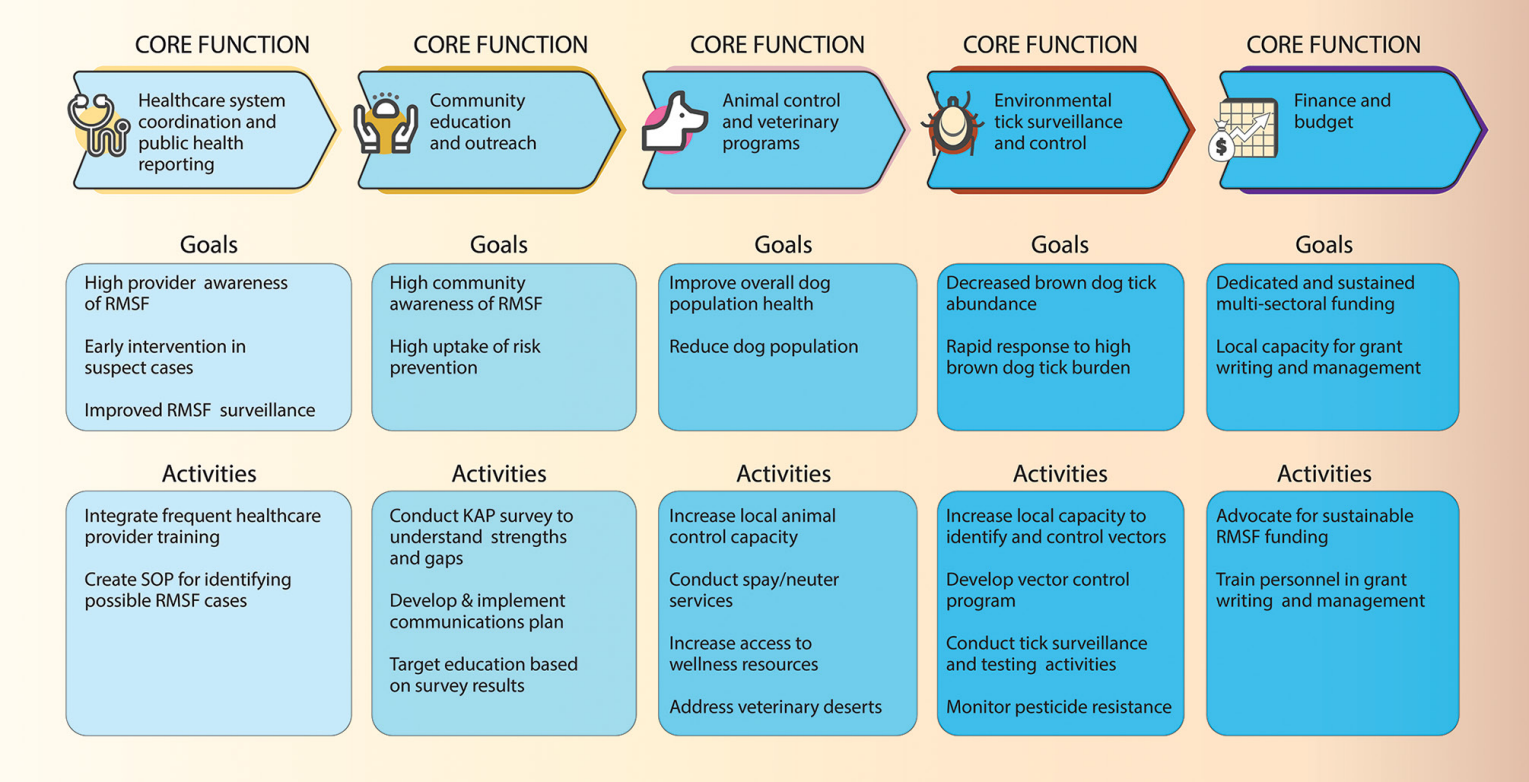
Recommended goals and activities for community-based prevention of brown dog tick–associated RMSF on the basis of the risk assessment road map for medium- or high-risk communities (Figure 1). KAP, knowledge, attitudes, and practices; RMSF, Rocky Mountain spotted fever; SOP, standard operating procedure.

Once leaders of a community have assessed its risk level, they should identify key stakeholders within their network and develop an action plan to address those risk factors and implement RMSF prevention before human cases occur (Table; Figure 2). Implementing an early warning system for rickettsial diseases can prevent illness and death among the human and canine populations and prevent high medical and indirect costs associated with RMSF (*25*,*26*). Activities to include in the action plan range from lower cost and effort, such as implementing standard operating procedures for identifying cases of RMSF in clinical settings, to high cost and effort, such as developing and maintaining a vector control program; feasibility and cost may depend on location and infrastructure. Communities should consider individual needs and resources to determine the level of RMSF risk response.

## Core Functions of RMSF Control

During the emergence of RMSF in Arizona, 5 core functions were identified as critical to prevent and control RMSF: 1) health care system coordination and public health reporting; 2) community education and outreach; 3) animal control and veterinary programs; 4) environmental tick control and surveillance; and 5) finance and budget. Increasing awareness of RMSF symptoms and treatment in the healthcare system and community might be among the most cost-effective interventions available to reduce RMSF illness and death because the bulk of the cost would be personnel time. However, interventions at the animal and environmental level are crucial to reducing tick populations and the potential for disease transmission.

### Function 1: Healthcare System Coordination and Public Health Reporting

In the United States, RMSF is a nationally notifiable condition within the spotted fever rickettsiosis (SFR) standard case definition, which captures diseases caused by multiple rickettsial agents (*27*). Whereas RMSF is effectively treated with antimicrobial drugs if they are given within 5 days of symptom onset, the nonspecific clinical manifestations can lead to misdiagnosis. Clinical illness is characterized by acute fever and may include headache, malaise, myalgia, nausea, and vomiting. The pathognomonic spotted petechial rash that often involves the palms or soles does not typically appear until after day 5 or 6 of illness. It is imperative that clinicians in medium- and high-risk communities are capable of recognizing, treating, and diagnosing RMSF to prevent severe illness and death. Healthcare providers should be trained to prophylactically begin doxycycline treatment and to order appropriate laboratory tests for diagnosis, including whole blood and plasma specimens for molecular tests and acute and convalescent serum specimens for indirect fluorescence antibody tests (*28*). In addition to clinical manifestations, diagnostic confirmation can be made on the basis of a 4-fold change in *R. rickettsii*–specific or SFR IgG titers by indirect fluorescence antibody with paired serum specimens or PCR confirmation of SFR DNA in an acute clinical specimen. Patient history should include questions about tick bites or direct contact with a tick-infested dog, ticks identified in or around the household, or travel to or residence in an area where RMSF cases have recently been identified. However, patients may not recall tick bites; therefore, failure to self-report a tick bite should not exclude a RMSF diagnosis. 

Our recommended indicators to assess risk level include the presence or absence of provider education around ticks and tickborne diseases, provider understanding of diagnosing and treating tickborne diseases, diagnostic testing capacity, distance to healthcare providers, and availability of medical transportation. We have identified many challenges in healthcare system coordination and public health reporting. Nonspecific symptoms may lead to misdiagnosis until the patient experiences critical clinical manifestations; laboratory testing is not always available, affordable, or expedient; PCR testing has low sensitivity in the acute stage of RMSF; serologic testing for detecting antibodies is frequently negative in the first week of illness, and the disease cannot be confirmed by using a single acute antibody result; patient loss to follow-up is high when a second visit to a healthcare provider is needed to collect a convalescent serum specimen, and the lack of convalescent titers has led to only 3% of SFR cases being reported as confirmed in the United States; surveillance may be limited when treatment is initiated on the basis of clinical manifestations and there is no laboratory confirmation of diagnosis; lack of case reporting reduces the ability of public health officials to conduct adequate surveillance and identify outbreaks; and misconceptions surrounding doxycycline administration to children persist, despite scientific studies showing its safety and efficacy.

### Function 2: Community Education and Outreach

Community members and stakeholders that are well informed about RMSF may be able to recognize the risk for brown dog tick–associated RMSF without knowing the infection rates in the brown dog tick or dog population. Some factors, such as fencing around property, conducting personal tick checks, and having community dogs spayed or neutered are protective against RMSF (*24*; M.K. Brophy, unpub. data). Other factors, such as solid waste or harborage near home and high dog density, especially free-roaming dogs, increase the risk for RMSF (*3*). Medium- and high-risk communities can prevent human illness and death from brown dog tick–associated RMSF by developing and implementing communications plans to inform the public about risk-mitigating factors. Public health outreach is more effective when tailored to the target population demographics (*29*–*32*). Communities should use locally minded verbiage and imagery and culturally relevant messaging and outreach through media with high uptake within the community.

Our recommended indicators to assess risk level include the percent of population below local poverty level, median years of educational attainment in community, presence or absence of health education around ticks and tickborne diseases, the community knowledge, attitudes, and behaviors surrounding personal and home tick prevention, and the percentage of the population with internet access. We have identified 3 challenges in community education and outreach. These challenges are the lack of culturally tailored educational materials, lack of staff to conduct outreach, and a low level of community resources to enable self-protective behaviors.

### Function 3: Animal Control and Veterinary Programs

Across the world, ≈75% of the >700 million domestic dogs are classified as free-roaming, or without human restraint or control (*33*). High densities of free-roaming dogs are associated with many public health concerns, including transmission of zoonotic diseases (*34*). Canine serosurveys have revealed ≈50%–60% of dogs in outbreak communities were IgG-positive for SFRs, indicating RMSF was circulating in the canine population before human cases were detected (*15*,*35*–*37*). Over time, a correlation between canine seroprevalence and RMSF cases and deaths was established (*38*,*39*). A compartment model to understand the dynamics of brown dog tick–associated RMSF was developed and discovered an ≈2-year lag between introduction of the pathogen to a naive canine population and epidemic-level transmission, further solidifying the need for early intervention (*40*).

Communities without veterinary services or animal control agencies might be more likely to have large populations of free-roaming dogs who have not been spayed or neutered or treated with tick preventatives, fostering an ideal environment for brown dog ticks to flourish. Dogs that travel between homes can transport *R. rickettsia–* infected ticks throughout a community, leading to the establishment of the bacterium in the tick population (*37*). Treating dogs with acaricidal products and promoting responsible pet ownership, including safely securing owned dogs on property, are critical activities to reduce tick population (*25*,*41*). Access to programs that provide veterinary care, spay and neuter, and adoption services can have a protective effect against RMSF transmission and should be considered an integral measure to protect human life.

We recommend multiple indicators to assess risk level. The first indicator is the wellness of the animal population, which includes the presence or absence of veterinary services, availability of effective ectoparasite treatments for dogs in the community, the number of spayed or neutered animals, and the cost of effective ectoparasite treatments for dogs in community. The second indicator we recommend is evaluating the free-roaming dog population status, which includes the density of free-roaming dogs, presence or absence of ordinances forbidding free-roaming dogs, fencing or tethering behaviors across the community, and the rate of brown dog tick infestation in dog population. The final indicator is community access to resources, which includes the presence or absence of an animal control department, presence or absence of animal shelter space, and the number of low- or no-cost spay and neuter clinics.

Several challenges are present in animal control and veterinary programs in medium and high-risk communities. Those challenges include a lack of affordable and available veterinary services, low community prioritization of animal wellness, no animal control ordinances or programs, and a limited availability of animal wellness supplies and treatments.

### Function 4: Environmental Tick Surveillance and Control

Whereas active surveillance of brown dog ticks and *R. rickettsii* is not feasible in most communities, if adequate vector control services are available, the burden of ticks on dogs and in the peridomestic environment can be loosely monitored. An increase in reports of ticks on dogs or around community homes could indicate increased risk for human infection.

Medium- and high-risk communities can use an integrative approach to prevent ticks by having solid waste removed and having homes treated with a properly applied acaricide in accordance with product labels. Two high-risk communities in Arizona conduct regularly scheduled pesticide application campaigns, with teams going door-to-door to apply pesticide around the perimeter of homes and estimate tick burden on dogs. Additional measures include treating dogs with acaricidal products and promoting responsible pet ownership because the canine hosts play a large part in the ticks’ ecology (*24*,*25*).

We recommend several indicators to assess risk level. The first is harborage, which includes the presence or absence of municipal or community solid waste removal and landfill cost and availability. The second indicator is pesticide use, which includes the presence or absence of vector control programs and the presence or absence of certified pesticide applicators. The final indicator is community knowledge, attitudes, and practices regarding tickborne diseases, which are influenced by the presence or absence of community education around ticks and tickborne diseases.

We have identified multiple challenges in environmental tick surveillance and control. Those challenges include the lack of vector control services specific to ticks, the lack of personnel trained in pesticide safety and application, the lack of solid waste removal, and the need for novel products and technologies for tick control.

### Function 5: Finance and Budget

Addressing risk factors for preventing and controlling brown dog tick–associated RMSF requires coordinated, sustained efforts to reduce the free-roaming dog population, increase community awareness, and reduce the number of ticks in the environment. However, because the response requires a multisectoral approach, adequate funding must be distributed across partners, including those not traditionally considered in disease prevention. A key strategy to ensuring the sustainability of prevention activities in medium risk or high-risk communities is to work across sectors to identify short- and long-term funding opportunities, including grant funding.

We recommend 2 key indicators for use in evaluating the financial and budget risk level of communities. The first is the presence or absence of dedicated annual jurisdictional funding to all partners for RMSF prevention. Second is the presence or absence of personnel capable of writing and submitting grant applications. 

We have identified 3 challenges to finance and budget security. First is the lack of sustainable funding, especially for tangential but necessary services. Second is the lack of infrastructure to house needed facilities. Last is the lack of financial commitment to ongoing prevention and control when the disease burden of RMSF is low.

## Discussion

Emerging infectious diseases, which are pathogens that have newly appeared, reappeared, or are rapidly increasing in incidence or range, are a global threat of high public health importance. More than 60% of emerging human diseases are zoonotic (*42*), affecting humans and animals alike. The effect of vectorborne zoonotic diseases on human health outpaces many other infectious diseases, warranting consideration of new and creative prevention and mitigation strategies (*43*). Whereas we cannot predict the specifics of an individual disease emerging, vectorborne zoonotic diseases such as RMSF will continue to emerge and are likely to expand in range, especially in the light of land-use and climate changes. Understanding the key transmission drivers and mitigation strategies is imperative to identifying high-risk areas of emergence and rapidly responding to protect human and animal health.

Certain factors associated with climate change, including increases in vector range and abundance, increases in wildlife and human interaction because of land use changes, and pathogen host shifting, are especially relevant to emerging vectorborne zoonotic diseases. The effect of climate change related temperature increases on brown dog tick range and density is unclear because the peridomestic tick can be present in high abundance year-round in some parts of the world. However, previous studies suggest the risk for humans being bit and contracting a disease from brown dog ticks may increase with higher temperatures (*44*,*45*). The resistance of this tick species to low humidities, high temperatures, and other environmental conditions that are considered unsuitable for most tick species is remarkable and will likely exacerbate challenges to control brown dog ticks as changes to climates continue (*23*,*45*,*46*).

Brown dog tick–associated RMSF is an emerging public health concern that can be prevented through proper assessment and action. Often it is unclear a community is at risk for endemic RMSF transmission until the first fatal human case occurs; however, there are clear instances when canine cases precede human cases, which demonstrates that preparedness and early detection before human cases are identified may save lives (*39*). The complexities of the RMSF transmission cycle indicate that the pathogen and risk are not likely to disappear any time soon. Maintaining vigilance and implementing integrated pest management strategies, including routine veterinary care and application of acaricides, in accordance with community risk level is crucial (*25*,*47*). The efforts to reduce tick populations and risk for RMSF transmission are cost- and labor-intensive endeavors that might prove unsustainable, especially in communities where access to resources are already restricted. Therefore, it is necessary to explore additional tools to add to the RMSF prevention toolbox, including novel prevention activities that are both scalable and sustainable, such as canine vaccine candidates against *R. rickettsii* or the brown dog tick itself (*48*). A vaccine might contribute greatly to reducing tick burdens on dogs or reduce the spread of *R. rickettsii* throughout the community.

Because of the interrelatedness of canine and human health regarding brown dog tick–associated RMSF, this complex One Health issue requires a communitywide multidisciplinary approach to reducing risk for disease in human and dog populations. Furthermore, the role of brown dog ticks in RMSF transmission is highly correlated with poverty and other social vulnerabilities, bringing health equity into focus (*4*,*14*,*49*–*51*). Most communities with endemic brown dog tick–associated RMSF are veterinary deserts with little or no access to care in the immediate area, which requires pet owners to travel long distances to seek veterinary care. In addition, many such communities in the United States are also in areas with limited access to medical care because of the rural nature of the environment. Increasing healthcare and veterinary services and accessibility in low-income settings are crucial goals to address health equity issues, including reducing RMSF risk. Until those goals can be realized, medium- and high-risk communities much establish realistic and scalable responses to help reduce RMSF risk on the basis of their resources and infrastructure abilities, such as ensuring human and animal healthcare providers are up to date in recognizing and treating this deadly disease.
